# A pan-cancer perspective analysis reveals the prognostic significance of SLC7A11 in hepatocellular carcinoma

**DOI:** 10.3389/fonc.2025.1601140

**Published:** 2025-09-05

**Authors:** Yinxu Zhang, Dai Wang, Xiaoyang Chen, Yuxi Wang, Guangyu Zhang, Xiaomei Liu

**Affiliations:** ^1^ Department of Surgery, The First Affiliated Hospital of Jinzhou Medical University, Jinzhou, Liaoning, China; ^2^ Department of Oncology, The First Affiliated Hospital of Jinzhou Medical University, Jinzhou, Liaoning, China

**Keywords:** SLC7A11, hepatocellular carcinoma, tumor microenvironment, prognostic biomarker, ferroptosis

## Abstract

**Background:**

The cystine/glutamate antiporter SLC7A11 is critically involved in tumorigenesis andferroptosis regulation. However, its comprehensive role in hepatocellular carcinoma (HCC) prognosis and tumor immunity remains elusive.

**Methods:**

We evaluated SLC7A11 expression using immunohistochemistry (IHC) in a clinical HCC cohort and analyzed its prognostic significance. A pan-cancer analysis across The Cancer Genome Atlas (TCGA) datasets was performed to investigate SLC7A11 genetic alterations, its impact on the tumor microenvironment (TME), tumor mutation burden (TMB), and therapy response.

**Results:**

SLC7A11 was significantly upregulated in HCC tissues, and high expression predicted poorer overall survival. Pan-cancer analysis confirmed its aberrant expression and identified a recurrent missense mutation (c.G>A) across various cancers. Elevated SLC7A11 expression correlated with higher TMB and TIDE scores, indicating a potential link to immunosuppression. It was enriched in metabolic pathways and associated with increased M0 macrophage infiltration. Furthermore, SLC7A11 expression positively correlated with response to agents including Gemcitabine and Bortezomib but conferred resistance to Cisplatin and others.

**Conclusion:**

Our findings establish SLC7A11 as a powerful prognostic biomarker for HCC, unveil its role in shaping an immunosuppressive TME, and highlight its value in predicting response to conventional and emerging therapies, providing a rationale for targeting SLC7A11 in future treatment strategies.

## 1 Introduction

Hepatocellular carcinoma (HCC), as the main type of primary liver cancer, is the sixth most frequent cancer affecting roughly 840,000 cases each year and leading to more than 780,000 deaths (1, 2). For early-stage HCC, surgical resection is still the primary treatment. For advanced-stage patients, locoregional and systemic therapies are the current treatment strategy, including targeted therapies, radiotherapy, immunotherapy, and transarterial chemoembolization (3, 4). According to clinical data, the average 5-year survival rate is 19.6%, while the rate can be reduced to 2.5% for advanced-stage HCC. However, the disease is usually diagnosed at the advanced stages, rendering locoregional and systemic therapies ineffective. Current surveillance relies on ultrasound and biomarkers such as alpha-fetoprotein (AFP)/des-gamma-carboxy prothrombin (DCP) via algorithms; however, their sensitivity remains limited in AFP-negative cases.

SLC7A11, also known as xCT, is a subunit of the cystine–glutamate antiporter, which is a key molecule that induces ferroptosis. Ferroptosis, as a newly recognized form of regulated cell death, participates in multiple pathological and physiological processes, such as T-cell immunity, acute renal failure, and cancer cell death (5). An upregulated SLC7A11 expression in tumor cells could accumulate cystine, triggering disulfide stress, and disulfidptosis presents a new insight into the mechanisms of tumor cells (6, 7). Numerous researchers have put forward that SLC7A11 is closely related to the initiation, proliferation, invasion, treatment, and prognosis of various tumors, including colorectal cancer, adrenocortical carcinoma, lung cancer, and ovarian cancer (8–11). Notably, previous data showed that ferroptosis mediated by SLC7A11 could effectively eliminate HCC cells (12). However, the exact molecular mechanisms and the functional value of SLC7A11 in HCC remain to be fully elucidated.

In this study, the specific function of SLC7A11 in HCC prognosis prediction and the molecular mechanisms involved were investigated. The expression levels of SLC7A11 in HCC were explored both in clinical tissue and in an online database. The SLC7A11 expression level and its mutation signature in pan-cancer were also explored, as well as the functional enrichment associated with SLC7A11 and its related genes. Moreover, data from The Cancer Genome Atlas (TCGA) were utilized to investigate the effect of SLC7A11 expression on the tumor microenvironment (TME), the tumor mutation burden (TMB), and the drug sensitivity.

## 2 Materials and methods

### 2.1 Clinical samples

#### 2.1.1 Patient data collection

This study retrospectively included data from 84 patients with HCC who met the inclusion criteria between 2020 and 2022, all of whom were newly diagnosed with HCC. Each patient had complete clinicopathological and follow-up data. The research involving human participants was approved by the Ethics Committee of the First Affiliated Hospital of Jinzhou Medical University under approval no. IIT-2024-807. This study was conducted in accordance with the ethical principles outlined in the Declaration of Helsinki, as established by the World Medical Association. Informed consent was obtained from all participants involved in the study. Clinicopathological variables including gender, age (≥60 years), TNM stage, the Eastern Cooperative Oncology Group (ECOG) performance status, smoking history, comorbidities (e.g., diabetes, hypertension, and coronary artery disease), the best response to first-line therapy, overall survival (OS), the SLC7A11 expression levels, and the survival status were all collected. Patients who did not undergo regular follow-up, as well as patients with mixed pathological types of HCC, were excluded. The sample size was determined based on power calculation using prior studies (HR=2.5, *α*=0.05, *β*=0.8), which estimated a minimum of 80 patients required to detect significant survival differences. This retrospective study included 84 patients, slightly exceeding the calculated threshold to enhance statistical robustness.

#### 2.1.2 Immunohistochemistry and scoring

The staining intensity and the percentage of stained cells were evaluated according to established reference standards. SLC7A11 expression was scored based on the staining intensity and the proportion of positive cells. Scoring was independently assessed by two pathologists in a double-blind manner, with discrepancies resolved by consensus. To ensure objectivity, the immunohistochemistry (IHC) slides were quantified using ImageJ software to measure the staining intensity and the positive cell proportion, and the final score was calculated as the average of the assessments of the two pathologists. For digital IHC analysis, whole-slide scans were processed in ImageJ v1.53 using color deconvolution to isolate 3,3′-diaminobenzidine (DAB) staining. The positive pixel percentage was calculated via automated thresholding (Otsu method). The digital scores correlated strongly with the pathologist assessments (*r*=0.91, *p* < 0.001). The final results were based on predefined evaluation criteria: no staining (0), weak staining (1), moderate staining (2), and strong staining (3). Based on the percentage of stained cells, the samples were categorized into two groups: low expression (0–1, ≤25%) and high expression (2–3, >25%).The 25% cutoff for IHC was clinically optimized via receiver operating characteristic (ROC) analysis [area under the curve (AUC) = 0.79] to reflect protein-level disease relevance, while median-based thresholds for transcriptomic data are standard for RNA sequencing (RNA-seq) survival analyses (Tang et al., 2017). This dual approach aligns with modality-specific standards: protein localization requires cellular context thresholds, whereas RNA-seq benefits from population-level cutoffs. Future multi-omics studies should harmonize criteria. For bioinformatic analyses using public datasets (e.g., TCGA), the mRNA expression levels of SLC7A11 were categorized into a high and a low group using the median expression value as the threshold (i.e., greater than or equal to the median = high expression; less than the median = low expression). The threshold was determined based on a combination of median expression levels (28%) and optimal ROC curve analysis (using the OS status) to ensure that it reflects clinically significant differences in the SLC7A11 expression (AUC =0.79, *p* < 0.001). This threshold has also been consistently applied across all analyses involving clinical samples and bioinformatics datasets (where the median expression was used to define the high/low groups).

#### 2.1.3 Screening of independent prognostic factors for HCC

Univariate Cox regression analysis was performed using the survival package (version 2.41-1) in R 4.1.2 (13) to assess the factors associated with HCC survival time. Covariates were selected for inclusion into the multivariate Cox regression model if they had established clinical relevance to HCC prognosis and/or demonstrated significant associations (*p* < 0.1) in the univariate analysis. Based on these criteria, the following covariates were included in the initial multivariate model: the Barcelona Clinic Liver Cancer (BCLC) stage, the cirrhosis status, the SLC7A11 expression level, the AFP level (≥400 *vs*. <400 ng/ml), the tumor diameter (≥5 *vs*. <5 cm), and comorbidities (e.g., diabetes, hypertension, hyperlipidemia, and cardiovascular disease). The final multivariate model was constructed using backward stepwise selection, with variables retained in the model based on the likelihood ratio test (*p* < 0.05).

#### 2.1.4 Statistical analysis

To analyze the correlation between SLC7A11 expression and clinicopathological characteristics, data with a normal distribution were assessed using independent samples *t*-tests. The Cox proportional hazards model was enrolled to perform multivariate analysis of the survival factors. A *p* < 0.001 was defined as the threshold for statistical significance. All statistical analyses were conducted using GraphPad Prism (version 9.5) and SPSS (version 27.0).

### 2.2 Analysis based on data from TCGA

#### 2.2.1 SLC7A11 expression levels in pan-cancer

To analyze the expression of SLC7A11 in pan-cancer, data from TCGA were utilized. Notably, the threshold for defining high/low expression in the bioinformatics datasets (the median mRNA expression) differed from that in clinical IHC samples (a 25% positive cell ratio), reflecting the distinct measurement modalities (transcriptional levels *vs*. protein localization and cellular proportion). The expression levels of SLC7A11 in various types of tumors and their corresponding controls were screened using Gene Expression Profiling Interactive Analysis (GEPIA) (14). Subsequently, the expression data for HCC were downloaded from TCGA database and displayed. To evaluate the diagnostic role of SLC7A11, an ROC curve was constructed using pROC (version 1.12.1) (15).

#### 2.2.2 Analysis of the mutation signature of SLC7A11

##### 2.2.2.1 SLC7A11 mutation signature based on the cBioPortal database

Information on SLC7A11 was screened from cBioPortal (https://www.cbioportal.org/) (16), and the mutation information of the gene in multiple types of cancers was further obtained.

##### 2.2.2.2 Mutation analysis based on maftools

The occurrence of tumors is closely related to gene mutations. Point mutations, small fragment deletions, and insertions could cause synonymous, missense, termination, and frameshift mutations in codons, then resulting in the loss of related functions of protein expression due to sequence changes. Ultimately, this leads to cellular malignancy and proliferation and, further, to the occurrence of tumors.

Mutation information on multiple tumors was downloaded from TCGA Genomic Data Commons (GDC) (https://portal.gdc.cancer.gov/repository), and the TMB of each tumor was calculated and displayed. The TMB difference between the high and low SLC7A11 expression levels in HCC was analyzed using the Wilcoxon test. The mutation status and the gene location of SLC7A11 were analyzed and displayed using maftools (version 2.6.05) (17) in R4.3.1.

#### 2.2.3 Functional analysis enriched by SLC7A11-related genes

Genes related to SLC7A11 were screened using the online software GeneMANIA (http://genemania.org) (18). Thereafter, the Gene Ontology (GO) and Kyoto Encyclopedia of Genes and Genomes (KEGG) pathways were investigated using DAVID (version 6.8) (19), with *p* < 0.05 as the threshold. GO contained three items: biological process (BP), molecular function (MF), and cellular component (CC).

#### 2.2.4 KEGG and HALLMARK pathways related to SLC7A11 based on GSEA

The KEGG and HALLMARK pathways were downloaded from Gene Set Enrichment Analysis (GSEA) (http://software.broadinstitute.org/gsea/index.jsp) (20), and the pathways related to the expression levels of SLC7A11 were screened based on the data from TCGA. In the analysis, three key statistical factors were calculated: the enrichment score (ES), the normalized enrichment score (NES), and the nominal *p*-value. In general, the functional gene sets with higher NES and lower nominal *p*-value have higher credibility. A *p* < 0.05 was considered as the threshold for the KEGG pathway selection.

#### 2.2.5 Immune-related analysis

##### 2.2.5.1 Microenvironment analysis

The tumor immune microenvironment refers to the proportion of immune cells in the tumor tissue. The proportions of different types of immune cells in the HCC tumor samples from TCGA were analyzed using CIBERSORT (https://cibersort.stanford.edu/index.php) (21) based on whole-genome expression levels. Thereafter, between the high and the low SLC7A11 expression group, differences in the immune cell distribution were compared using the Wilcoxon test. Subsequently, the ESTIMATE score, the immune score, the stromal score, and the tumor purity of the HCC tumor samples in TCGA were calculated using the estimate package (https://bioconductor.org/packages/release/bioc/html/estimate.html) (22) in R4.1.2. Differences in the tumor purity, stromal score, immune score, and ESTIMATE score between groups with low and high SLC7A11 expression levels were also compared. Finally, the relationship between the expression level of SLC7A11 and the infiltration of immune cells was calculated with the ESTIMATE score using the cor function in R 4.1.2.

##### 2.2.5.2 Response to immune checkpoint therapy

The response of the HCC patients in TCGA to immune checkpoint therapy was evaluated using Tumor Immune Dysfunction and Exclusion (TIDE; http://tide.dfci.harvard.edu/) (23) based on the whole-genome expression level of the HCC samples in TCGA, which was represented by the TIDE score. The response type of each patient to treatment was then returned. Afterward, the Wilcoxon test was used to compare the differences in the TIDE scores of the high and low SLC7A11 expression groups, and the correlation between the TIDE scores and the SLC7A11 expression levels was also calculated. A detailed explanation of how immune evasion and the response to checkpoint blockade are functionally linked to SLC7A11 expression has been added. This includes a more in-depth analysis of the correlation between SLC7A11 expression levels and specific immune checkpoint pathways, as well as the potential mechanistic role of SLC7A11 in modulating the immune responses within the TME.

#### 2.2.6 Drug sensitivity analysis

The sensitivity of each patient to chemical drugs was estimated using pRRophetic (https://bioconductor.org/packages/release/bioc/html/pRRophetic.html) (14) in R4.3.1 based on the expression levels of the genes involved in the HCC samples in TCGA combined with Genomics of Drug Sensitivity in Cancer (GDSC; https://www.cancerrxgene.org/) (24). In the analysis, the IC_50_ value represents drug sensitivity. The Wilcoxon test was used to compare the differences in the IC_50_ values of the drugs between the high and low SLC7A11 expression groups, and the correlation between the SLC7A11 expression level and the IC_50_ value was also calculated. The pRRophetic IC_50_ estimates reflect the cell line responses and may not fully capture the *in vivo* pharmacokinetics or the tumor heterogeneity.

## 3 Results

### 3.1 Upregulated SLC7A11 in HCC and poorer prognosis were observed in clinical samples

#### 3.1.1 Overexpression of SLC7A11 in HCC based on IHC

**Figure 1:**
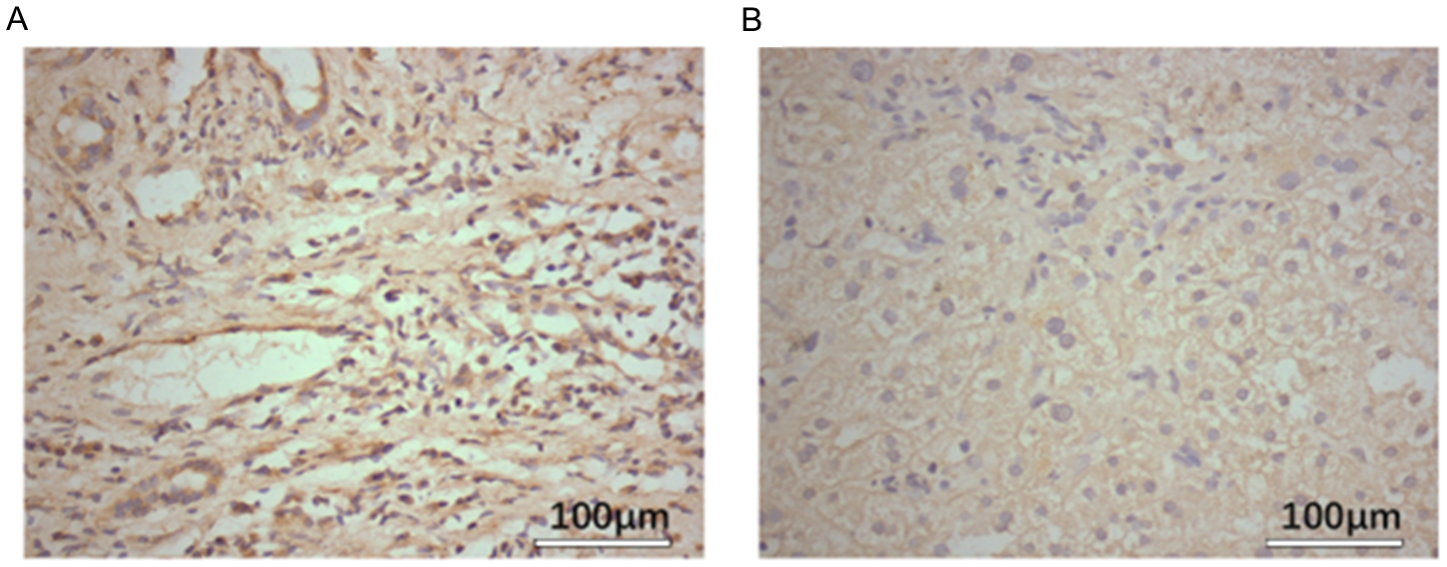
*SLC7A11* expression in hepatocellular carcinoma (HCC) tissues is higher than that in normal tissues. **(A)** Expression levels of SLC7A11 in tumor tissue. **(B)** Expression levels of SLC7A11 in adjacent non-cancerous tissues.

The expression level of SLC7A11 was examined using IHC. Notably, SLC7A11 expression was higher in HCC cells compared with adjacent normal tissues, with positive immunostaining localized primarily to the cytoplasm of HCC cells. As shown in **Figure 1**, the SLC7A11 expression in normal adjacent tissues was ≤25%, indicating low expression, while that in liver cancer tissues was >25%, indicating high expression. The inter-rater reliability for IHC scoring was evaluated using Cohen’s kappa coefficient, which demonstrated excellent agreement (*κ*=0.85, *p* < 0.001).

#### 3.1.2 Clinicopathologic characteristics of 84 HCC patients

A detailed analysis of the baseline characteristics of the 84 patients with HCC is shown in **Table 1**: of the patients, 26.2% were aged more than 60 years, 89.3% were men and 10.7% women, 94.0% had an ECOG performance status of 0–1, 36.9% had hypertension, 38.1% had diabetes, 38.1% had coronary artery disease, 22.6% had hyperlipidemia, 42.9% had liver cirrhosis, and 47.6% had an upregulated SLC7A11 level. The first-line treatment included targeted therapies with sorafenib and lenvatinib, with or without the immunosuppressants programmed cell death protein 1 (PD-1)/programmed death-ligand 1 (PD-L1) inhibitors. Efficacy was assessed based on the follow-up results using the RECIST v1.1 criteria for solid tumors, with the best response recorded. After first-line treatment, the overall response rate was 95.3%, with 34 patients (40.5%) achieving partial response (PR) and 46 patients (54.8%) achieving stable disease (SD). Among the 84 patients, 40 patients (47.6%) had higher SLC7A11 expression, while 44 patients (52.4%) had a low expression.

**Table 1:** Clinicopathological features of the patients with hepatocellular carcinoma (HCC).

Clinical characteristic	*n* (%)
Age (years)	
≥60	22 (26.2%)
<60	62 (73.8%)
Gender	
Men	75 (89.3%)
Women	9 (10.7%)
ECOG score	
0–1	79 (94.0%)
≥2	5 (6.0%)
AFP (ng/ml)	
≥400	44 (52.4%)
<400	40 (47.6%)
Hypertension	
Yes	31 (36.9%)
No	53 (63.1%)
Diabetes	
Yes	32 (38.1%)
No	52 (61.9%)
Cardiovascular disease	
Yes	32 (38.1%)
No	52 (61.9%)
Hyperlipidemia	
Yes	19 (22.6%)
No	65 (77.4%)
Tumor diameter	
≥5 cm	48 (57.1%)
<5 cm	36 (42.9%)
*SLC7A11* expression level	
High	40 (47.6%)
Low	44 (53.4%)
Cirrhosis	
Yes	36 (42.9%)
No	48 (57.1%)
Efficacy of first-line therapy	
PR	34 (40.5%)
SD	46 (54.8%)
PD	4 (4.8%)

ECOG, Eastern Cooperative Oncology Group; *AFP*, alpha-fetoprotein; *PR*, partial response; *SD*, stable disease; *PD*, progressive disease.

#### 3.1.3 Overexpression of SLC7A11 had poorer survival performance

We compiled the clinical and pathological information from 84 cases and conducted both univariate and multivariate analyses to calculate the medians and *p*-values, assessing the correlation of various pathological features with HCC. Univariate analysis indicated that liver cirrhosis, the BCLC stage, and the SLC7A11 expression level are significant risk factors affecting prognosis in patients with HCC (*p* < 0.001) (**Table 2**). Higher SLC7A11 expression (HR = 3.338, 95%CI = 1.727–6.453) and BCLC staging (HR = 4.468, 95%CI = 2.499–9.467) are independent prognostic factors for HCC (*p* < 0.001), as revealed by the multivariate Cox regression analysis.

**Table 2:** Relationship between *SLC7A11* expression and the clinicopathological factors.

Factors	OS				
	Median	UVA	MVA		
	(months)	*p*	*p*	HR	95%CI
Age (years)					
≥60	51.14	0.141	0.283	1.619	0.672–3.899
<60	36.88				
Sex					
Men	39.50	0.347	0.903	1.090	0.272–4.369
Women	46.44				
SLC7A11					
Low	54.93	<0.001**	<0.001**	3.338	1.727–6.453
High	27.66				
AFP (ng/ml)					
≥400	31.48	0.010	0.140	1.702	0.840–3.450
<400	50.48				
Cirrhosis					
Yes	25.18	<0.001**	0.178	1.593	0.809–3.135
No	52.33				
ECOG					
0–1	40.10	0.514	0.168	0.233	0.030–1.845
2–3	37.00				
Diabetes					
Yes	40.74	0.997	0.250	0.687	0.362–1.303
No	39.01				
Hypertension					
Yes	40.32	0.951	0.183	1.562	0.810–3.013
No	40.60				
Hyperlipidemia					
Yes	42.53	0.775	0.455	1.346	0.618–2.935
No	39.99				
Cardiovascular disease					
Yes	34.74	0.208	0.626	1.177	0.611–2.269
No	44.08				
Pathological stage					
Stage I	35.50	0.312	0.141	1.602	0.855–3.002
Stage II	44.22				
Stage III	34.13				
Tumor diameter					
≥5 cm	31.96	0.005*	0.241	0.570	0.223–1.458
<5 cm	52.37				
BCLC					
0 or A	58.14	<0.001**	<0.001**	4.468	2.499–9.467
B	26.63				
C or D	6.78				

*UVA*, univariate analysis; *MVA*, multivariate analysis; ECOG, Eastern Cooperative Oncology Group; *AFP*, alpha-fetoprotein; *BCLC*, Barcelona Clinic Liver Cancer.

*p < 0.01; **p < 0.001.

**Figure 2:**
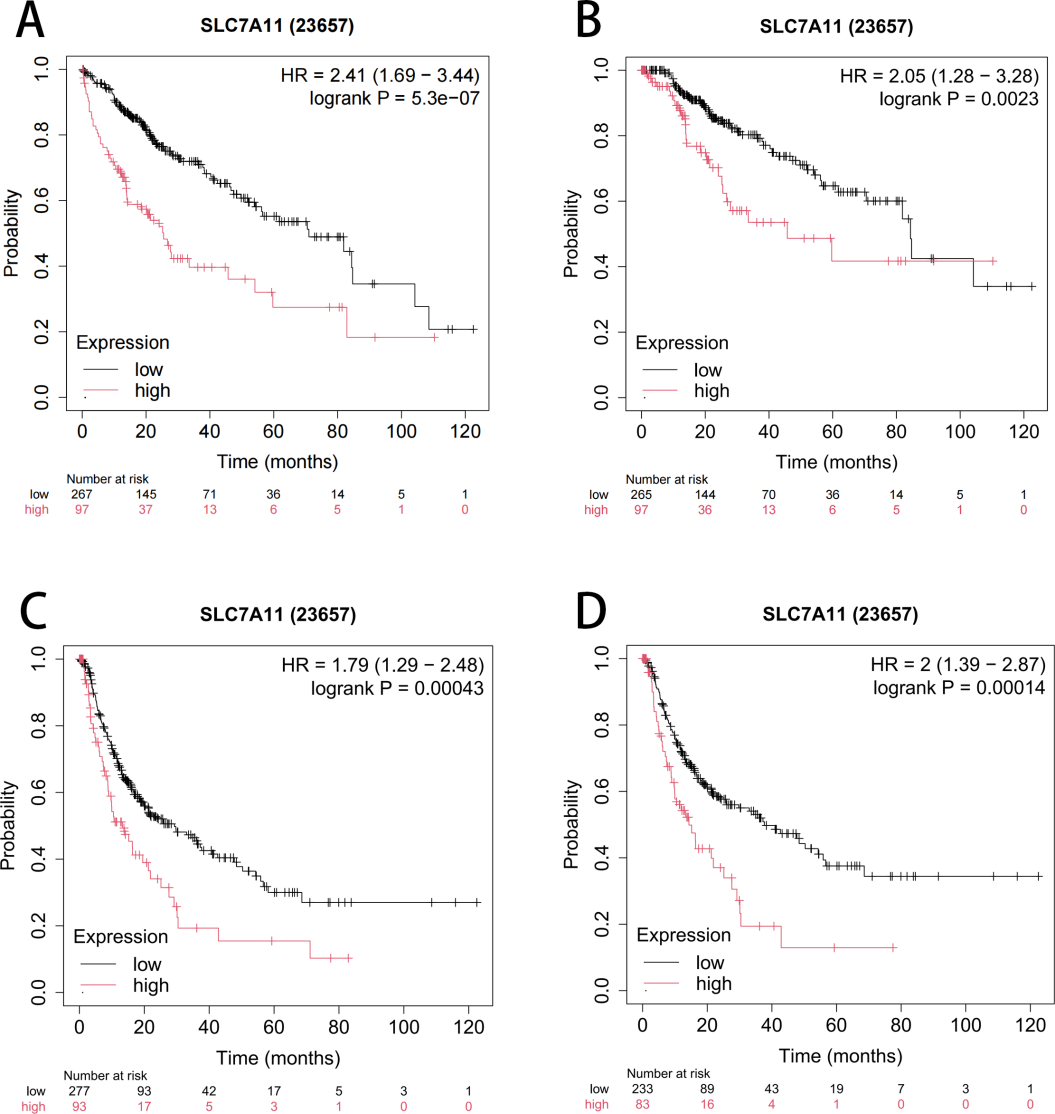
Kaplan–Meier curve assessing the impact of SLC7A11 expression on overall survival (OS) **(A)**, progression-free survival (PFS) **(B)**, relapse-free survival (RFS) **(C)**, and disease-specific survival (DSS) **(D)**.

Furthermore, the impact of SLC7A11 overexpression on the prognosis of HCC was assessed using the Kaplan–Meier plotter (KM-plotter). As shown in **Figure 2**, the results indicated that the upregulation of SLC7A11 is correlated with a significantly reduced OS (HR = 2.41, 95%CI = 1.69–3.44, *p* = 5.3e−7), progression-free survival (PFS) (HR = 2.05, 95%CI = 1.28–3.28, *p* = 0.0023), relapse-free survival (RFS) (HR = 1.79, 95%CI = 1.29–2.48, *p* = 0.00043), and disease-specific survival (DSS) (HR = 2.41, 95%CI = 1.39–2.87, *p* = 0.00014). These findings suggest that the overexpression of SLC7A11 in HCC correlates with poorer patient prognosis.

### 3.2 Expression level and mutation signature of SLC7A11 in pan-cancer

#### 3.2.1 SLC7A11 was upregulated in multiple types of cancers and related to poorer overall survival

**Figure 3:**
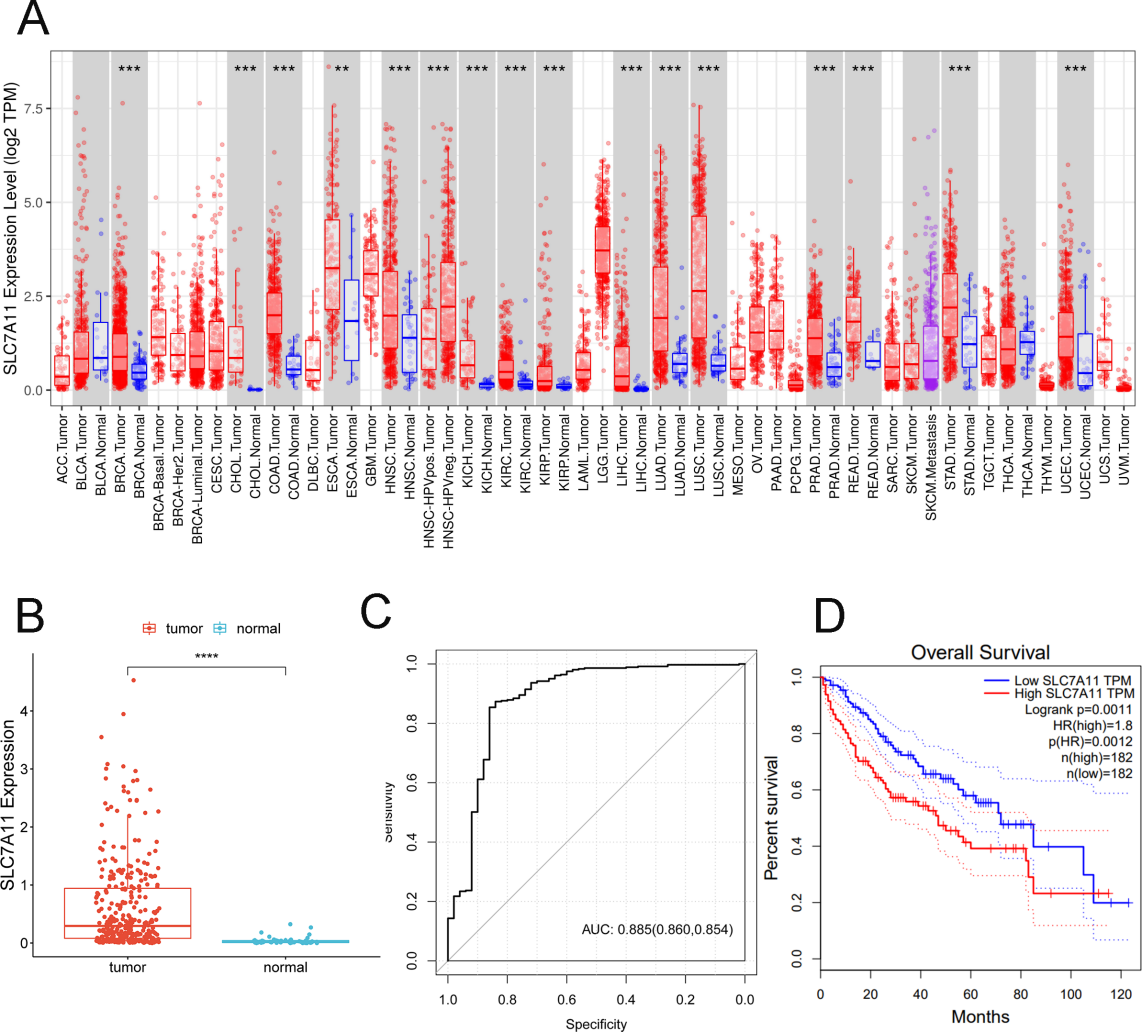
SLC7A11 is upregulated in multiple types of cancers and is related to poorer overall survival. **(A)** Expression levels of SLC7A11 in 38 tumor types. **(B)** Expression levels of SLC7A11 in liver cancer. **(C)** Receiver operating characteristic curve analyzing the ability of SLC7A11 expression in distinguishing the types of liver cancer. **(D)** Kaplan–Meier curve analyzing survival in the high and low *SLC7A1* expression groups. ***p < 0.001.

Based on the data from GEPIA, significantly higher SLC7A11 levels were observed in various types of cancers, including breast invasive carcinoma (BRCA), cholangiocarcinoma (CHOL), colon adenocarcinoma (COAD), head and neck squamous cell carcinoma (HNSC), kidney chromophobe (KICH), kidney renal clear cell carcinoma (KIRC), lung adenocarcinoma (LUAD), lung squamous cell carcinoma (LUSC), prostate adenocarcinoma (PRAD), rectum adenocarcinoma (READ), stomach adenocarcinoma (STAD), and uterine corpus endometrial carcinoma (UCEC) (**Figure 3A**). Thereafter, the expression levels of SLC7A11 in HCC and normal controls were obtained from TCGA. The data showed that the expression levels of SLC7A11 were significantly higher in HCC (*p* < 0.0001) (**Figure 3B**). Furthermore, the AUC of the ROC analysis of the diagnostic ability of SLC7A11 was 0.885 (0.860, 0.854), suggesting its valuable diagnostic ability in HCC (**Figure 3C**). The KM curve demonstrated that upregulated SLC7A11 levels had poorer OS (**Figure 3D**). External validation was performed using the independent GEO dataset GSE117570 (HCC cohort). The ROC analysis for distinguishing HCC from adjacent normal tissue yielded an AUC of 0.862 (95%CI = 0.832–0.891). This result closely mirrors our TCGA-based AUC (0.885) and strongly supports the diagnostic potential of SLC7A11. Median-based stratification is standard for transcriptomic data, but may not capture nonlinear biological effects.

#### 3.2.2 SLC7A11 mutation in pan-cancer based on the cBioPortal and GDC databases

**Figure 4:**
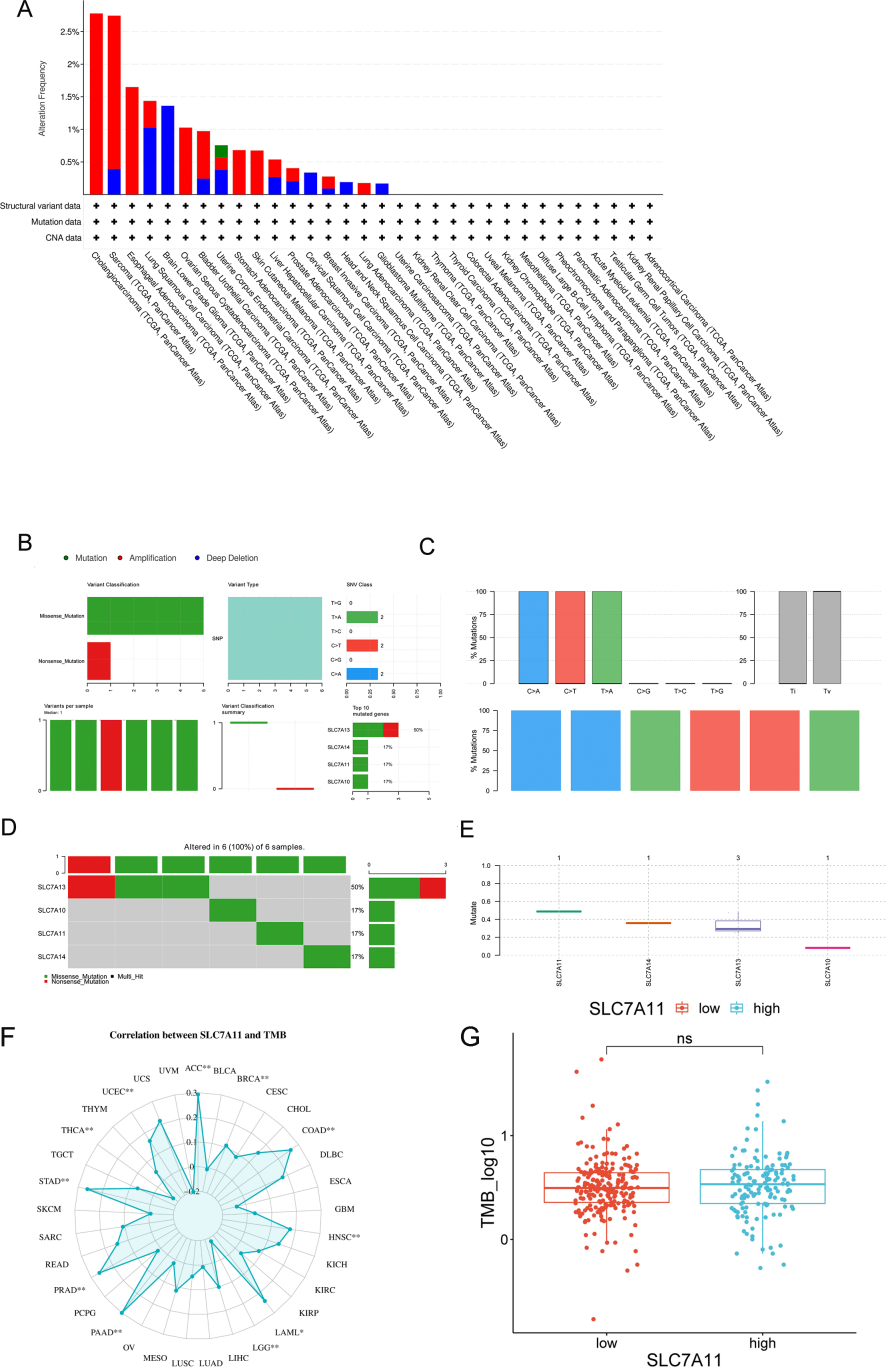
Characteristics of SLC7A11 mutations in pan-cancer based on the cBioPortal and GDC databases. **(A)** SLC7A11 mutations in 32 tumor types based on the cBioPortal database. **(B–E)** Mutations of *SLC7A10*, SLC7A11, *SLC7A13*, and *SLC7A17* in liver cancers. **(F)** Analysis of the correlation between SLC7A11 expression and tumor mutation burden (TMB) across 33 cancer types in TCGA database. **(G)** Comparison of the TMB values between different *SLC7A11* expression levels.

SLC7A11 mutations in multiple types of cancers were screened in the cBioPortal database. **Figure 4A** shows the two main types of mutations in SLC7A11 observed: 26.88% homdel and 26.88% amp. Based on the GDC database, we observed the same missense mutation (G>A) in SLC7A11 and its related gene families in HCC (**Figures 4B–E**). **Figure 4E** specifically illustrates the distribution of this missense mutation in the SLC7A11 coding region, highlighting its potential impact on protein structure. Furthermore, the association between TMB and SLC7A11 expression level in pan-cancer was further investigated. **Figure 3F** shows a significant association between TMB and SLC7A11 expression level in multiple types of cancers, including CAAD, adrenocortical carcinoma (ACC), COAD, HNSC, PRAD, and STAD. The association between TMB and SLC7A11 expression was also observed in liver hepatocellular carcinoma (LIHC). TMB analysis revealed that a high SLC7A11 expression was associated with an increased TMB across multiple cancer types, including HCC. **Figure 4G** displays a box plot comparing the TMB values between the high and low SLC7A11 expression groups in HCC, demonstrating a significantly higher TMB in the high expression group (*p* < 0.001).

#### 3.2.3 Interaction network and functional enrichment of SLC7A11 and its 20 related genes

**Figure 5:**
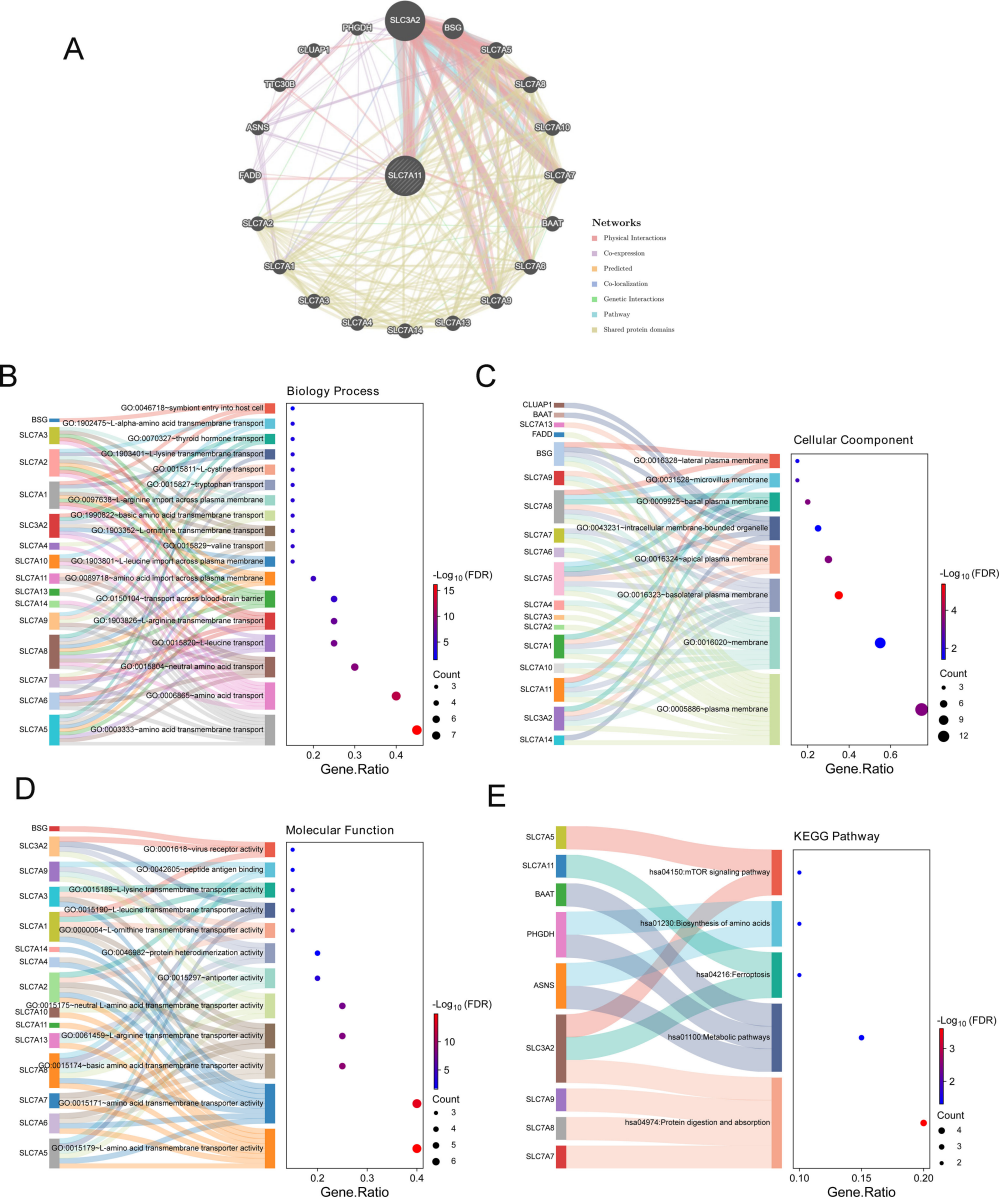
Functional enrichment and networks enriched by genes related to SLC7A11. **(A)** Interaction network of SLC7A11 and its related genes based on GeneMANIA. **(B–D)** Gene Ontology (GO) biological process (BP) **(B)**, GO molecular function (MF) **(C)**, and GO cell component (CC) **(D)** enriched by SLC7A11 and its related genes. **(E)** Kyoto Encyclopedia of Genes and Genomes (KEGG) pathways enriched by SLC7A11 and its related genes.

Based on the GeneMANIA database, 20 genes related to SLC7A11 were screened out (**Figure 5A**). Furthermore, the results of the functional enrichment showed 18 GO BP terms (**Figure 5B**), including “GO: 0006865 amino acid transport,” “GO:0003333 amino acid transmembrane transport,” and “GO: 0015804 neutral amino acid transport,” eight GO CC terms (**Figure 5C**), 12 GO MF terms (**Figure 5D**), and five KEGG pathways (**Figure 5E**), including “hsa04974: Protein digestion and absorption” and “hsa01100: Metabolic pathways,” which were significantly enriched by SLC7A11 and its 20 related genes.

#### 3.2.4 KEGG and HALLMARK pathways enriched by SLC7A11 based on GSEA

Based on the expression levels of the whole genome of HCC in TCGA and the GSEA algorithm, 12 KEGG pathways (**Supplementary Figure S1A**), including “KEGG_UBIQUITIN_MEDIATED_PROTEOLYSIS,” “KEGG_GLYCINE_SERINE_AND_THREONINE_METABOLISM, “ and “KEGG_LYSOSOME,” and eight HALLMARK pathways (**Supplementary Figure S1B**), including “HALLMARK_WNT_BETA_CATENIN_SIGNALING,” “HALLMARK_PROTEIN_SECRETION,” and “HALLMARK_MTORC1_SIGNALING,” were significantly enriched by SLC7A11.

#### 3.2.5 The expression level of SLC7A11 could affect the immune microenvironment and the response to immune treatment

##### 3.2.5.1 Immune microenvironment analysis

**Figure 6:**
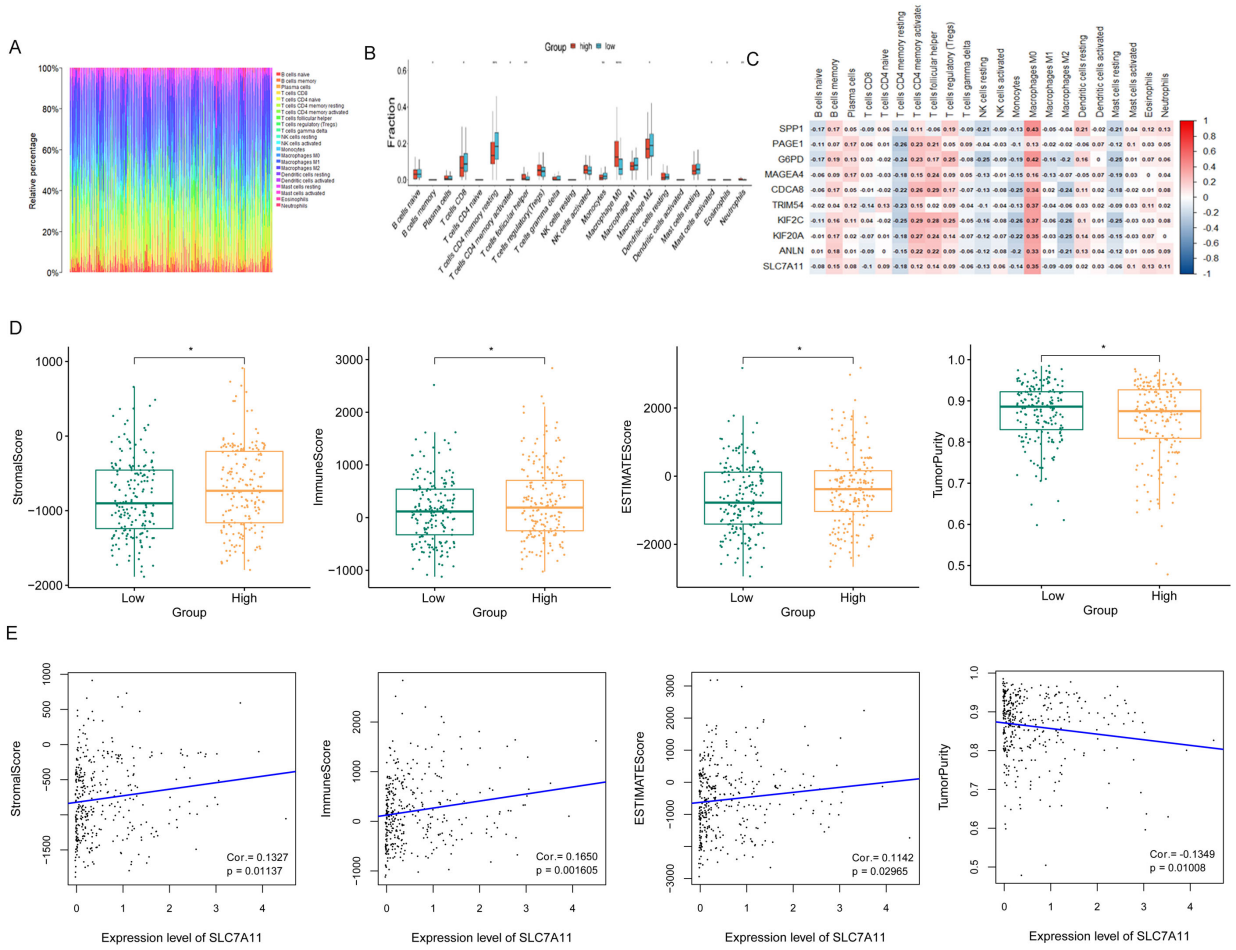
Immune microenvironment performance related to SLC7A11 expression. **(A)** Ratio of immune cells involved in hepatocellular cancer. **(B)** Proportions of 22 immune cell types in samples with high and low SLC7A11 expression levels. The results showed that seven immune cell subtypes were more prevalent in the high SLC7A11 expression group, while eight subtypes were more prevalent in the low expression group. **(C)** Heatmap of immune cells and related genes. **(D)** Stromal score, immune score, ESTIMATE score, and tumor purity comparisons between the high and low SLC7A11 expression groups. **(E)** Correlation between the ESTIMATE score and the expression level of SLC7A11.

The ratios of immune cells in cancer patients based on CIBERSORT are shown in **Figure 6A**. Furthermore, the ratios of 22 immune cells in the high and low SLC7A11 expression groups were compared, with the results showing seven types of immune cells being more prevalent in the high SLC7A11 expression group, including regulatory T cells (Tregs), neutrophils, resting dendritic cells, activated natural killer (NK) cells, plasma cells, T follicular helper cells, and M0 macrophages. Eight immune cells were more prevalent in the low SLC7A11 expression group, including resting memory CD4 T cells, M1 macrophages, CD8 T cells, naive B cells, gamma delta T cells, monocytes, M2 macrophages, and resting mast cells (**Figure 6B**). Furthermore, the genes significantly related to these immune cells were explored. It was observed that, compared with other immune cells, the expression level of SLC7A11 was positively related to the infiltration of M0 macrophages, with a closer relationship (**Figure 6C**). M0 macrophage infiltration showed the strongest positive correlation with SLC7A11 expression (*r* =0.35, *p* < 0.001) (**Figure 6C**). This diagnostic accuracy was further validated in an independent cohort (GSE117570), where the AUC for SLC7A11 was 0.823 (95%CI = 0.781–0.865), supporting the robustness of its predictive value (**Figure 3D**).

In order to assess the signature of the microenvironment, the ESTIMATE scores of the high SLC7A11 group *versus* the low SLC7A11 group were compared. **Figure 6D** shows that samples with high SLC7A11 expression have higher stromal scores, immune scores, and ESTIMATE scores, suggesting the more complex immune microenvironment in samples with high SLC7A11 expression. The SLC7A11 level was positively related to the stromal score, the immune score, and the ESTIMATE score and negatively related to tumor purity (**Figure 6E**).

##### 3.2.5.2 SLC7A11 may serve as a biomarker for immune checkpoint therapy

**Figure 7:**
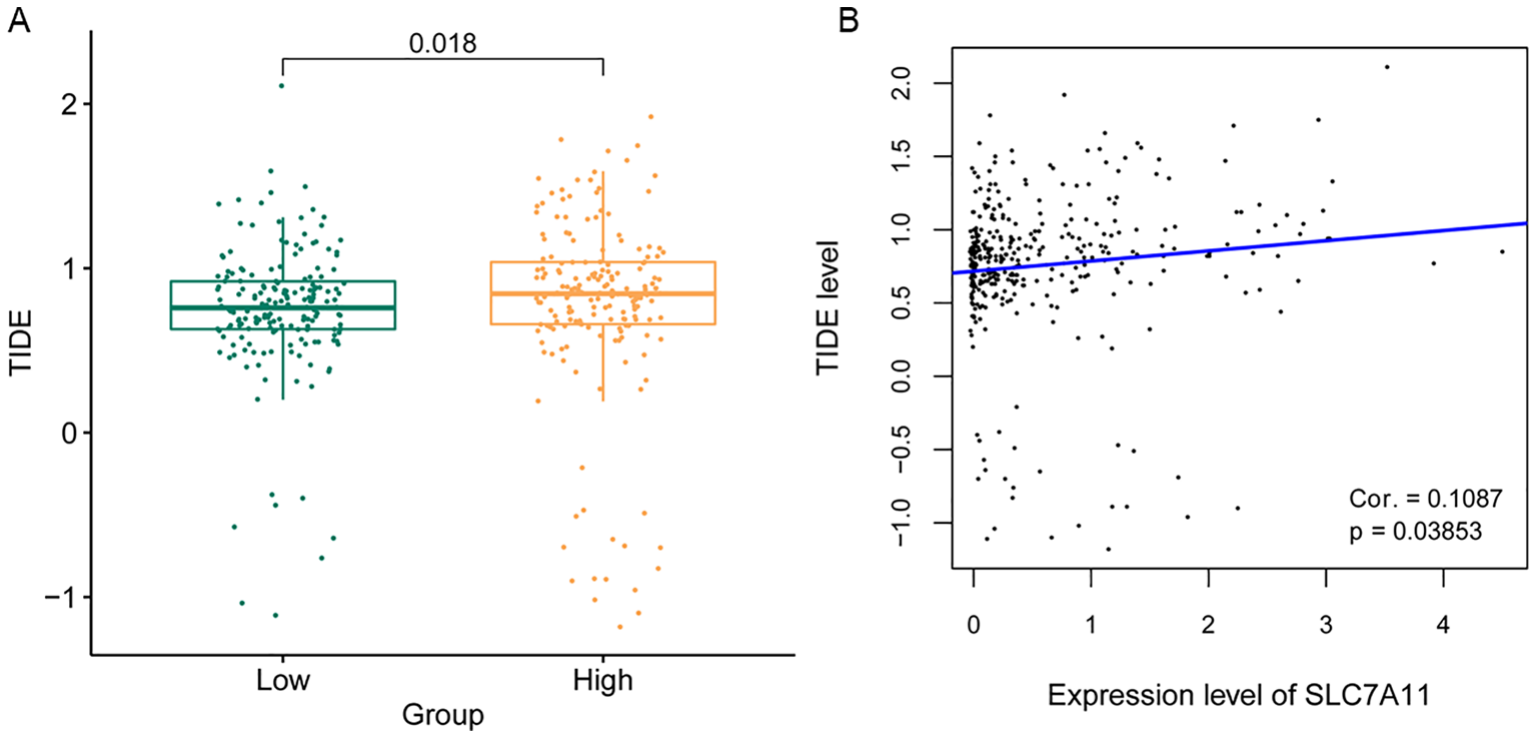
Analysis of the sensitivity to immune checkpoint therapy according to the SLC7A11 expression level. **(A)** Tumor immune dysfunction and exclusion (TIDE) score comparison between high and low SLC7A11 levels. **(B)** Correlation between the TIDE score and the expression level of SLC7A11.

A patient’s response to immune checkpoint therapy was predicted using the TIDE database. As shown in **Figure 7A**, the high SLC7A11 expression group had higher TIDE values, suggesting that SLC7A11 may potentially influence immune evasion by influencing T-cell dysfunction and exclusion. The correlation between the SLC7A11 expression level and the TIDE value was further calculated. The SLC7A11 expression level showed a significant positive correlation with the TIDE value (**Figure 7B**), but requires mechanistic validation.

#### 3.2.6 Drug sensitivity analysis

The sensitivity of each patient to chemical drugs was estimated based on the expression levels of the genes involved in the HCC patients in TCGA and the relevant small-molecule drugs obtained from the GDSC database. Thereafter, the IC_50_ values of 19 small-molecule drugs were obtained. Furthermore, the performance of these 19 drug small molecules in the high and low SLC7A11 expression groups was compared. A significant difference was observed in nine small-molecule drugs: AZD6482, AZD7762, bortezomib, cisplatin, erlotinib, gefitinib, gemcitabine, nilotinib, and vinorelbine (**Supplementary Figure S2A**). Subsequently, the association between these nine small-molecule drugs and the SLC7A11 level was further investigated, which showed that the SLC7A11 level was positively related to four types of small-molecule drugs (i.e., gemcitabine, AZD7762, bortezomib, and vinorelbine) and negatively related to five types of small-molecule drugs (i.e., AZD6482, cisplatin, erlotinib, gefitinib, and nilotinib) (**Supplementary Figure S2B**).

## 4 Discussion

Recently, the occurrence of HCC has steadily increased, with tumor invasion and metastasis being the main factors contributing to the high recurrence rate and the poor prognosis in advanced HCC (25). Currently, AFP is the most widely used biomarker in HCC; however, AFP-negative cases have been observed in many patients with advanced HCC, highlighting the urgent need for new biomarkers that can complement AFP to enable the early diagnosis of HCC and to improve patient survival rates (26). The prognostic independence of SLC7A11 from AFP (**Table 2**) suggests its utility in AFP-negative subgroups, potentially enhancing the existing biomarker frameworks. In the present study, significantly upregulated SLC7A11 levels were confirmed in HCC tissue and multiple types of cancers, which were related to poorer survival. While our correlative data suggest that SLC7A11 may promote immune evasion via TIDE-associated mechanisms, functional validation in immune–tumor co-culture models is needed to establish causality. In HCC, samples with upregulated SLC7A11 had higher TMB and TIDE values, and the gene was mainly enriched in transport-related biological processes and metabolism-related pathways and positively related to the infiltration of M0 macrophages. The expression level of SLC7A11 was also positively related to the stromal score, the immune score, and the ESTIMATE score, as well as to four types of small-molecule drugs (i.e., gemcitabine, AZD7762, bortezomib, and vinorelbine), while the gene was negatively related to tumor purity and to five types of small-molecule drugs (i.e., AZD6482, cisplatin, erlotinib, gefitinib, and nilotinib). Thus, we propose that the expression level of SLC7A11 may help predict the survival of cases with HCC and that the level of which, alongside AFP, could offer deeper insights into the progression of HCC. SLC7A11 complements AFP by identifying high-risk AFP-negative HCCs. Integrating SLC7A11 into the existing algorithms (e.g., GALAD score) could enhance early detection, as demonstrated by Shahini et al. (2023). Future studies should evaluate combinatorial panels (AFP+SLC7A11) to enhance early detection.

SLC7A11, also known as xCT, which is a sodium-independent 12-pass transmembrane transporter protein, is widely expressed in tissues such as the liver and the brain. SLC7A11 was demonstrated as a key molecule mediating ferroptosis, and ferroptosis is a unique type of cell death induced by a disruption in the iron metabolism and the antioxidant system imbalance. Numerous studies have also demonstrated that multiple cancer cells are sensitive to ferroptosis via SLC7A11 by mediating tumor suppressors such as ATF4, NRF2, and TP53 (27). Previous evidence has shown that the expression level of SLC7A11 may be a potential biomarker for HCC diagnosis and prognosis (12). In the present study, both IHC and the findings from TCGA supported an upregulated SLC7A11 in HCC. Multivariate analyses of the clinicopathological factors showed that SLC7A11 expression is an independent risk factor for HCC, and KM showed that patients with overexpression of SLC7A11 show increasing trends for poorer survival. In the pan-cancer analyses, SLC7A11 was highly expressed in solid tumors, with its upregulation associated with worse prognosis, suggesting that it may serve as a novel prognostic biomarker (28). External validation in the ICGC cohort confirmed the prognostic significance of SLC7A11, reinforcing its clinical translatability. Functional enrichment showed that the gene was mainly enriched in transport-related biological processes and metabolism-related pathways. Growing evidence pointed to metabolism dysregulation in the pathogenesis of HCC, such as glutamine, amino acid, and fatty acid metabolism (29). Thus, we propose that SLC7A11 may be a potential tumor-promoting gene in HCC.

Numerous factors are related to HCC development, including alcohol use, trace elements, and hepatitis B virus (HBV) (30). For most patients, HCC arises from a progression from hepatitis to cirrhosis. Currently, for HCC treatment, the efficacy of targeted therapies and immunotherapy has been well demonstrated, and identifying new therapeutic targets could help delay disease progression and offer alternative treatment options. To assess the response sensitivity to immunotherapy, the immune microenvironment plays a decisive role in the prognosis and treatment outcomes (31), in particular infiltrating immune cells (32). In HCC, SLC7A11 expression correlated with the increased infiltration of M0 macrophages and elevated TIDE scores, suggesting an association with immunosuppressive microenvironments. These correlations nominate SLC7A11 for experimental validation in immune-evasion models. While these findings align with *in vitro* observations of SLC7A11-mediated CD8^+^ T-cell suppression, mechanistic studies are needed to establish causality. Further mechanistic studies, such as SLC7A11 knockdown in immune cell–tumor co-cultures, are warranted to validate these associations. Zhang et al. previously constructed a risk model based on M0 macrophages and their related genes, with the model showing a promising role in predicting prognosis and therapeutic responses (33). Moreover, samples with upregulated SLC7A11 had higher TMB and TIDE values, and the expression level of SLC7A11 was positively related to the stromal score, the immune score, and the ESTIMATE score, while the gene was negatively related to tumor purity. Currently, a high TMB is often associated with an enhanced immune responsiveness (34). SLC7A11 is correlated with immunosuppressive TME features, including M0 macrophages and elevated TIDE scores. This observation aligns with emerging evidence that SLC7A11/xCT-mediated cystine metabolism modulates immune activity. Elevated cystine uptake and glutamate release can deplete the extracellular cystine/cysteine, limiting T-cell activation and function. Increased extracellular glutamate can suppress T-cell responses via glutamate receptors and promote Treg activity. Driving the antioxidant production (e.g., glutathione) potentially protects tumor cells from immune-derived ROS and confers resistance to T-cell killing. A high SLC7A11 activity, by altering the cystine/glutamate levels and the redox state, could favor an immunosuppressive M2-like polarization. Further investigation into the specific role of SLC7A11 in modulating macrophage function and T-cell activity within the HCC TME is warranted.

Some limitations should be noted. Notably, this study has several limitations. Firstly, the functional link between SLC7A11 and immune evasion was inferred from correlational analyses (e.g., TIDE score and macrophage infiltration) rather than mechanistic experiments. Secondly, the precise mechanisms by which SLC7A11 contributes to HCC should be explored in further investigations. Thirdly, the diagnostic accuracy of SLC7A11 (AUC=0.885) was derived from TCGA data and lacked validation in an external cohort, which may affect the generalizability of the findings. Future studies with independent multicenter datasets are needed to confirm these results.

In conclusion, our study demonstrated that SLC7A11 is upregulated in HCC and that its overexpression is associated with poor prognosis. Notably, SLC7A11 showed prognostic independence from AFP, suggesting that it could enhance HCC risk stratification when incorporated into surveillance algorithms alongside AFP and DCP. Current HCC screening and monitoring algorithms, such as the GALAD score, rely heavily on AFP and DCP. However, these algorithms have limitations, particularly in AFP-negative cases, where sensitivity remains a challenge. For instance, the GALAD score, which combines AFP, DCP, age, and gender, has shown high accuracy in HCC risk assessment, but may miss a subset of patients with low AFP levels. SLC7A11, with its diagnostic accuracy (AUC=0.885), offers a promising avenue to complement the existing biomarkers. Its ability to identify high-risk AFP-negative HCC patients could significantly enhance the sensitivity of surveillance protocols. Previous studies have explored the integration of novel biomarkers into existing frameworks. For example, research highlighted in PMID: 36901717 discussed the potential of combining novel markers with AFP and DCP to improve early detection (35). Similarly, SLC7A11 could be integrated into algorithms such as the GALAD score, potentially improving surveillance accuracy. The correlations of SLC7A11 with immune evasion signatures also warrant further experimental validation to assess its therapeutic relevance in immunotherapy resistance. However, the precise mechanisms by which SLC7A11 contributes to HCC development and its functional link to immune evasion require further investigation. Future studies with independent, multicenter datasets are necessary to confirm these results and to establish SLC7A11 as a reliable tool in clinical practice.

## Data Availability

The datasets presented in this study can be found in online repositories. The names of the repository/repositories and accession number(s) can be found in the article/**Supplementary Material**.

## Supplementary material

Supplementary Figure 1 Pathway enrichment analysis of SLC7A11 in HCC. **(A)** KEGG pathways significantly enriched in high SLC7A11 expression group (nominal P<0.05, NES>1.5). **(B)** HALLMARK pathways associated with SLC7A11 expression (nominal P<0.05, NES>1.3). NES, normalized enrichment score; FDR, false discovery rate.

Supplementary Figure 2 Drug sensitivity analysis of SLC7A11 in HCC. **(A)** IC50 value differences of nine small molecular drugs between high and low SLC7A11 groups (Wilcoxon test, P<0.05). **(B)** Correlation between SLC7A11 expression and drug IC50 values (Spearman’s rank correlation). Positive correlation indicates higher drug resistance with increased SLC7A11 expression.

Supplementary Table 1 Dataset for all HCC.
