# Supplementary figures and images for: A pan-cancer perspective analysis reveals the prognostic significance of SLC7A11 in hepatocellular carcinoma
Source: Front Oncol. 2025 Sep 5;15:1601140. doi: 10.3389/fonc.2025.1601140 (PMC12447273; doi:10.3389/fonc.2025.1601140)

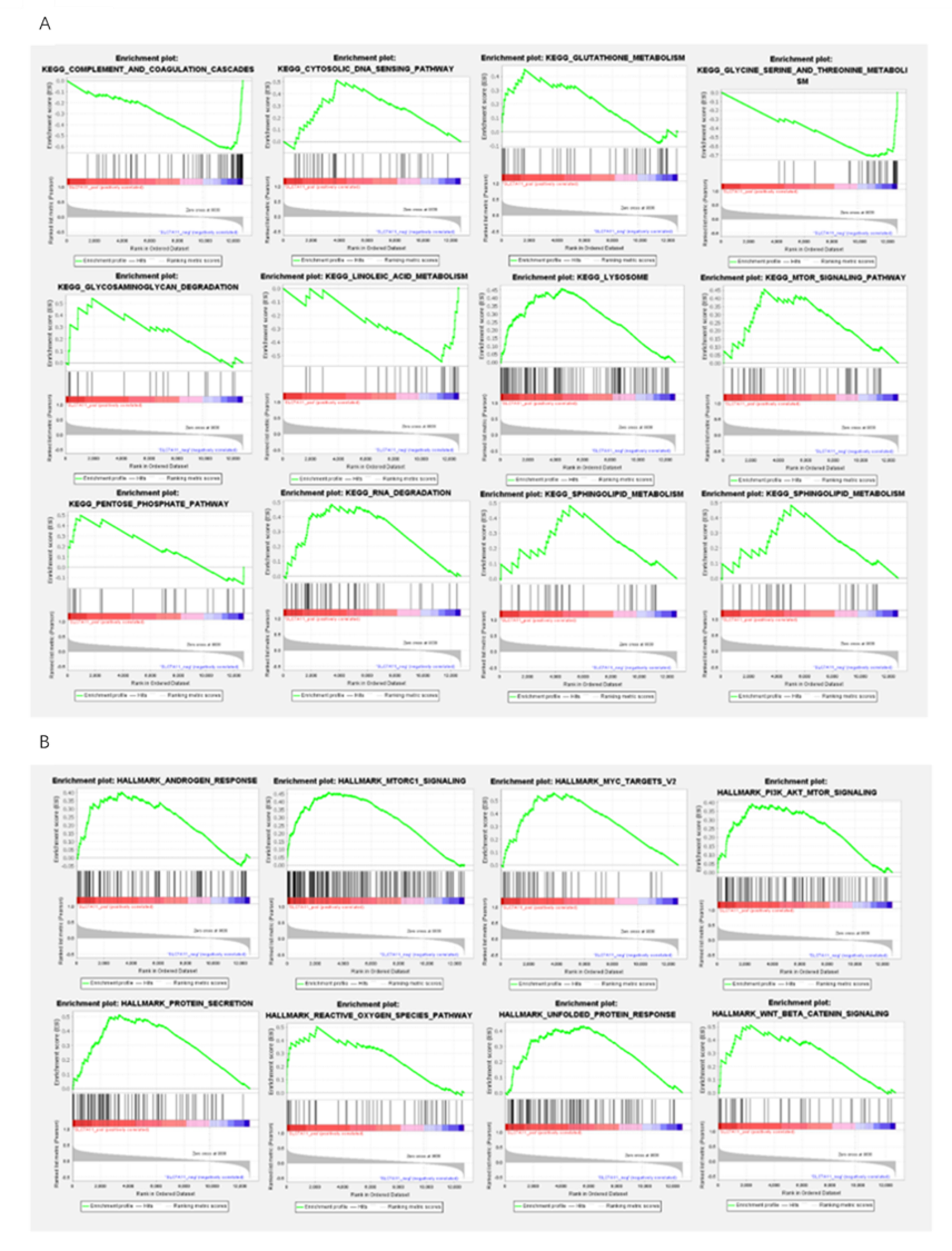

Supplement: Supplementary Figure 1 — Pathway enrichment analysis of SLC7A11 in HCC. (A) KEGG pathways significantly enriched in high SLC7A11 expression group (nominal P<0.05, NES>1.5). (B) HALLMARK pathways associated with SLC7A11 expression (nominal P<0.05, NES>1.3). NES, normalized enrichment score; FDR, false discovery rate. [file Image1.tif]

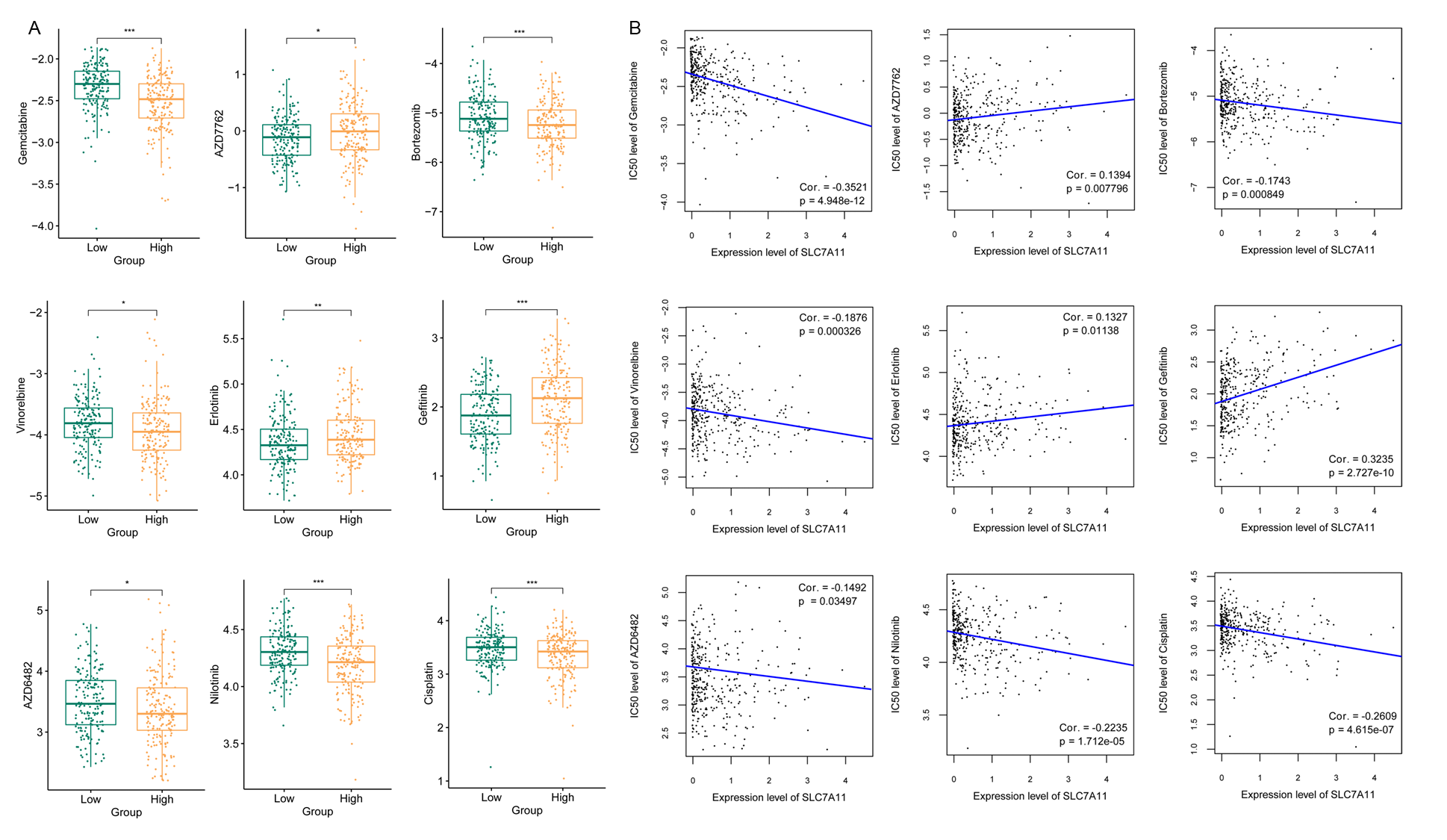

Supplement: Supplementary Figure 2 — Drug sensitivity analysis of SLC7A11 in HCC. (A) IC50 value differences of nine small molecular drugs between high and low SLC7A11 groups (Wilcoxon test, P<0.05). (B) Correlation between SLC7A11 expression and drug IC50 values (Spearman’s rank correlation). Positive correlation indicates higher drug resistance with increased SLC7A11 expression. [file Image2.tif]
